# Dietary Intake of Micro- and Nanoplastics: Potential Adverse GI Effects on Microbiome, Inflammation, and Neoplasia

**DOI:** 10.3390/microorganisms14061309

**Published:** 2026-06-11

**Authors:** Michael Saadeh, Gordon Hong, Sana Rabeeah, Priyata Dutta, Edward C. Oldfield, David A. Johnson

**Affiliations:** 1 Department of Internal Medicine, University Hospitals Cleveland Medical Center, Cleveland, OH 44113, USA; michael.saadeh@uhhospitals.org (M.S.); gordon.hong2@uhhospitals.org (G.H.); 2 Department of Internal Medicine, University of Toledo, Toledo, OH 43606, USA; sana.rabeeah@utoledo.edu; 3 Department of Internal Medicine, Trinity Health, Ann Arbor, MI 48197, USA; priyatadutta576@gmail.com; 4 Sentara Gastroenterology Specialists, Norfolk, VA 23510, USA; ecoldfield@gmail.com; 5 Division of Gastroenterology, Macon and Joan Brock Virginia Health Sciences at Old Dominion University, Norfolk, VA 23507, USA

**Keywords:** microplastics, nanoplastics, gastrointestinal tract, gut microbiome, dysbiosis, intestinal barrier, mucosal immunity, inflammatory bowel disease, colorectal cancer

## Abstract

Micro- and nanoplastics (MNPs) are pervasive in food-contact environments and the human diet, positioning the gastrointestinal (GI) tract as the primary portal of entry and a plausible site of early biological effects. Human exposure is supported by detection of microplastics in stool and colon tissue, and emerging clinical studies report associations between fecal microplastic burden and GI disease states, including inflammatory bowel disease (IBD) and colorectal cancer (CRC). Preclinical studies provide mechanistic plausibility, reporting that ingested MNPs can modulate microbial ecology, alter mucus membrane integrity, increase intestinal permeability through changes in cellular tight junction biology, and induce inflammatory gene expression. These effects can vary by MNP polymer type, particle size/shape, aging state, and exposure dose. Human-relevant experimental platforms increasingly demonstrate size- and concentration-dependent uptake and host responses while revealing substantial inter-individual variability. We synthesize current evidence on dietary sources and key physiochemical properties as they relate to mechanistic pathways connecting MNP exposure to dysbiosis–immune activation–neoplasia axes, in addition to methodological limitations that constrain current clinical utility. Further research including standardized biomonitoring and exposure protocols, environmentally realistic chronic low-dose mixtures, longitudinal human cohorts, and interventional designs that test whether exposure reduction modifies GI inflammation biomarkers and cancer-relevant pathways are critical to clarifying causality.

## 1. Introduction

Exposure to microplastics (MPs) and nanoplastics (NPs), defined as plastic particles <5 mm and <100 nm, respectively, is increasingly recognized as a biologically relevant phenomenon, reflecting widespread environmental presence of plastics, food-chain contamination, and food-contact release pathways [1,2]. Diet is frequently treated as a dominant exposure pathway as plastics have been reported in drinking water, seafood and other foods, and food-contact contexts, though occurrence estimates vary with detection thresholds and analytical workflows and are incomplete for many staple food groups [3]. Approximately 15% of caloric intake could be associated with ingestion of up to ~52,000 micro- and nanoplastic (MNP) particles annually, and true totals could be substantially higher due to limited data across many major food groups and methodological limitations [3]. The gastrointestinal (GI) tract is a compelling focus as it is the first sustained interface with ingested particles. Notably, the GI mucosal ecosystem integrates barrier function, immune function, and the gut microbiome, which can convert persistent perturbations into chronic inflammation or pro-neoplastic signaling [4]. Human biomonitoring supports exposure and GI contact: microplastics have been detected in human stool and in colectomy tissues, motivating careful investigation of local mucosal interactions and potential downstream disease associations [5]. This review focuses on sources and physiochemical determinants of dietary micro- and nanoplastics (MNPs) and the potential impact of MNPs on the gut microbiome and intestinal barrier health, GI neoplasia, and translational impacts.

### Literature Search Strategy

This narrative review was primarily conducted through a systematic search of PubMed using the following terms and combinations: “microplastics,” “nanoplastics,” “micro- and nanoplastics,” “gut microbiome,” “dysbiosis,” “intestinal barrier,” “inflammatory bowel disease,” “colorectal cancer,” “intestinal inflammation,” “dietary exposure,” and “GI neoplasia.” No formal date restriction was applied. Additional references were identified through review of reference lists from retrieved articles and from author expert knowledge of the field. Given the narrative nature of this review, formal bibliometric analysis was not performed; the included studies were selected to provide a representative and clinically relevant synthesis of current evidence across preclinical, in vitro, and human research. All authors contributed to reference identification and selection.

## 2. Dietary MNP: Sources, Characteristics, and Gastrointestinal Effects

### 2.1. Dietary Sources and Exposure Pathways

Dietary contamination can occur during production, harvesting, processing, packaging, and storage [2]. Seafood and drinking water have historically dominated the occurrence literature, but experts caution that relative contributions are unresolved and likely population- and behavior-dependent, with important but undermeasured contributions from atmospheric deposition to foods, food preparation, and packaged foods [3].

Official guidance emphasizes uncertainty rather than definitive risk estimates: the World Health Organization technical document on microplastics in drinking water summarizes known occurrence and treatment-removal evidence and highlights major knowledge gaps for health impact inference; the European Food Safety Authority statement similarly stresses insufficient toxicological and toxicokinetic data for human risk assessment, especially for NPs [6].

Vulnerable exposure contexts include infant feeding, where polypropylene infant feeding bottles can release very high microplastic particle counts during formula preparation under realistic temperature and handling scenarios [7]. These ubiquitous sources suggest that a consistent food intake is a homogeneous path of human MNP exposure [8].

### 2.2. Physicochemical Properties

Biological interaction depends not only on particle presence but on size distribution, shape (fibers/fragments/spheres), polymer identity, aging state, and surface chemistry—including additive content and sorbed contaminants [9]. Risk-assessment work argues that microplastics constitute a multidimensional “particle diversity” problem and that exposure and effects should be described in distributions and aligned using appropriate dose metrics (particle number, mass, surface area), which many studies do not report comparably [9].

From an analytical standpoint, reliable polymer identification and quantification requires validated particle-based (e.g., micro-FTIR/Raman) and/or mass-based (e.g., pyrolysis GC–MS) approaches with contamination control and recovery testing. The comprehensive review by Ivala also emphasized the sensitivity limits and challenges for detection of MNPs [10].

While MPs and NPs are frequently discussed together, NPs exhibit distinct toxicokinetic properties that warrant specific consideration. NPs are estimated to have significantly greater oral bioavailability compared to MPs [11]. Their small size confers substantially greater potential for cellular internalization, paracellular and transcellular epithelial crossing, and systemic distribution. As such, current evidence suggests that larger MPs may primarily disrupt extracellular biological interfaces and local tissue ecosystems, whereas NPs may act on an intracellular level [12]. Size-dependent uptake mechanisms include endocytosis through enterocytes, transcytosis via M-cells over Peyer’s patches, and paracellular diffusion, with smaller particles more likely to cross epithelial barriers [12]. Once absorbed, NPs have been shown to distribute systemically to the liver, lung, kidney, reproductive organs, and other tissues, raising concern about remote organ effects following GI exposure [12]. Furthermore, the clearance of NPs is likely physiologically distinct from MPs. Using a physiologically based toxicokinetic model with mice, Chen et al. demonstrates an association between particle size and urinary clearance, noting a marked increase in clearance rate with increased NP size [13]. NPs also present unique analytical challenges, as their detection requires specialized techniques and they remain substantially under characterized in human biomonitoring studies relative to MPs [12]. There is likely overlap in the pathophysiology of MPs and NPs given the spectrum of particle sizes; however, the current evidence base for NP-specific GI effects remains more limited than for MPs, and extrapolation between size classes should be made cautiously.

### 2.3. Gastrointestinal Transit, Retention, and Uptake

Most ingested particles involve GI transit and excretion, consistent with routine detection in fecal biomonitoring studies [5]. The GI transit, however, does not imply biological inertness. Notably, even without systemic absorption, luminal interactions with mucus, epithelial surfaces, and microbial communities can alter barrier and immune signaling [12]. Evidence supporting possible local retention includes the detection and characterization of MNPs in colectomy specimens, supporting the plausibility of tissue-level exposure in the colon and motivating investigation of local mucosal effects [14]. MNPs may traverse the intestinal barrier and enter into the systemic vascular circulation with widespread distribution to virtually all organs [15].

**Figure 1:**
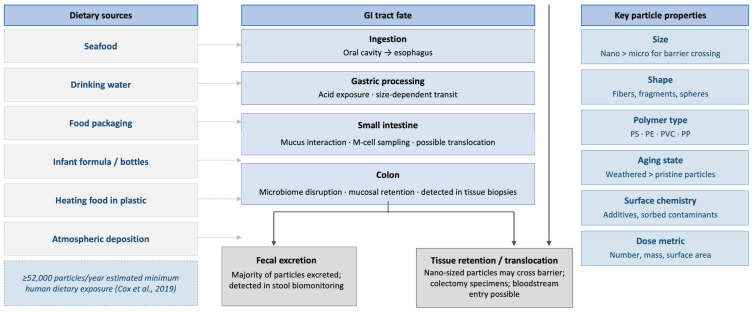
Dietary sources of micro- and nanoplastics (MNPs) and their fate in the gastrointestinal tract. Left panel: major dietary exposure pathways including seafood, drinking water, food packaging, infant feeding bottles, plastic food preparation, and atmospheric deposition, with estimated minimum human dietary intake of ≥52,000 particles per year [3]. Center panel: GI transit from ingestion through gastric processing, small intestinal interaction, and colonic exposure, with the majority of particles excreted in feces and nano-sized particles potentially entering tissues or the bloodstream. Right panel: key physicochemical properties modulating biological fate and effect. PS = polystyrene; PE = polyethylene; PVC = polyvinyl chloride; PP = polypropylene.

Mechanistic uptake hypotheses include sampling by M cells over Peyer’s patches and translocation facilitated by barrier disruption; the probability of uptake is expected to increase as particle size decreases. Therefore, current research emphasizes that NPs may be more likely to cross barriers than larger MPs under some conditions (Figure 1) [12].

## 3. Impact of MNPs on Gut Microbiome

Exposure to MNPs is associated with significant alterations to the gut microbiome with likely significant functional and metabolic consequences and related potential health implications. The existing literature suggests that MNPs are associated with a reduction in microbial diversity and shifts toward pro-inflammatory taxa. A study by Lu et al. exposed mice to varying sizes of polystyrene microplastic materials and subsequently evaluated gut microbiome composition [16]. Their study notably demonstrated lower levels of *Firmicutes* ssp. and α-*Proteobacteria* in stool samples of mice exposed to microplastics, as well as reduced diversity of the gut microbiome, identified by RNA sequencing from the cecum of exposed mice [16]. The *Firmicutes* phyla represents a significant portion of the healthy gut microbiome and is considered important in processing fiber and modulating inflammatory responses [17]. Another study using an in vitro colon model, meant to represent the human gut, also evaluated exposure to MNPs and subsequent changes in the intestinal microbiome. Their study found a rise in opportunistic bacteria associated with a reduction in bacterial flora considered to be beneficial [18].

Exposure to MNPs is associated with functional and metabolic consequences in the gut microbiome. Animal model studies suggest that dysbiosis, as a result of MNP exposure, leads to alterations in short-chain fatty acid (SCFA) production and SCFA-mediated signaling pathways [19]. SCFAs, such as acetate, propionate, and butyrate, are thought to be critical in many physiologic processes including maintenance of the gut barrier, modulating metabolism, and regulating inflammatory pathways [20].

MNPs may also affect the gut microbiota at the level of gene expression, including alterations related to antibiotic resistance [21]. Mouse model studies demonstrate shifts in the functional metagenome, the collective genes and their associated functions, of gut microbiota towards antibiotic resistance associated with MNPs [22]. A multicenter, cross-sectional study was conducted by Liu et al., evaluating microplastic concentration, microbiome composition, and metagenome sequencing of stool samples from preschool age children in China [23]. Their findings show that microplastic exposure may influence the makeup of the gut microbiome, as has been demonstrated in previous studies, but notably also suggest that exposure may have an impact on microbial functional pathways and may be associated with antibiotic resistance gene activity [23].

Bile acid metabolism is also thought to be impacted by MNP exposure. Bile acids are critical regulators of intestinal immune homeostasis and inflammatory signaling, with dysregulation implicated in both IBD pathogenesis and colorectal carcinogenesis. A mouse model study by Wen et al. sheds light on this phenomenon and potential mechanisms, demonstrating cholestasis in exposed mice as a result of impaired bile acid synthesis and alterations in bile acid efflux-related genes of the liver [24]. Furthermore, bile acid concentrations in stool are also notably different in mice exposed to microplastics, suggesting alterations in bile acid metabolism [24]. The impact of MNP exposure to the gut microbiome is concerning given the potential downstream effects. Further longitudinal studies are necessary to understand the long-term impacts of MNPs on human health and potential targets for therapeutic intervention (Table 1).

## 4. Microplastics, Intestinal Barrier Dysfunction, Immune Activation, and Chronic Inflammation

### 4.1. Epithelial Barrier and Mucus Layer Disruption

**Figure 2:**
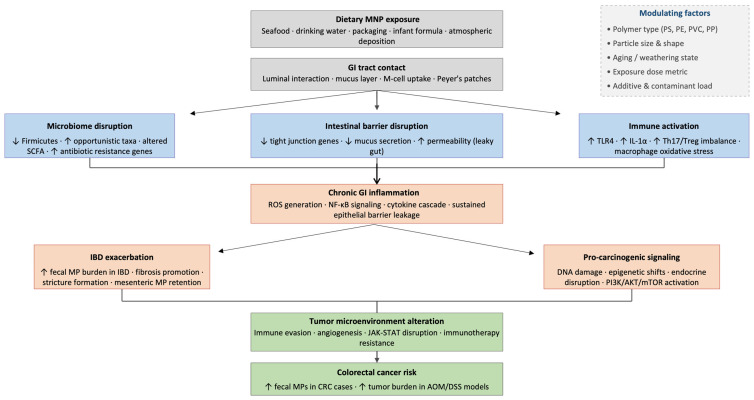
Proposed mechanistic pathway connecting dietary MNP exposure to gastrointestinal neoplasia. Following ingestion, MNPs contact the intestinal mucosa and converge on three parallel mechanisms: microbiome disruption (reducing beneficial taxa, altering SCFA production, and increasing antibiotic resistance gene activity), intestinal barrier dysfunction (reducing tight junction gene expression and mucus secretion, increasing permeability), and immune activation (upregulating TLR4, IL-1α, and Th17/Treg imbalance). These mechanisms drive chronic GI inflammation, which may promote IBD exacerbation or pro-carcinogenic signaling (DNA damage, epigenetic shifts, PI3K/AKT/mTOR activation), ultimately contributing to tumor microenvironment alteration and increased colorectal cancer risk. Effects are modulated by polymer type, particle size and shape, aging state, and exposure dose. ↑ indicates increase or upregulation; ↓ indicates decrease or downregulation. MNP = micro/nanoplastic; SCFA = short-chain fatty acid; ROS = reactive oxygen species; TLR4 = Toll-like receptor 4; IL-1α = interleukin-1α; IBD = inflammatory bowel disease; CRC = colorectal cancer.

Exposure to MNPs is ubiquitous, yet mechanisms linking exposure to disease are incompletely defined. Current evidence implicates intestinal barrier dysfunction, immune activation, and chronic inflammation as plausible contributors to microplastic-associated toxicity (Figure 2). Barrier dysfunction (“leaky gut”) is clinically relevant to GI diseases including IBD and celiac disease and is also implicated in diverse systemic inflammatory and metabolic conditions; thus, barrier endpoints are a key translational bridge for MNP research [31].

### 4.2. Innate and Adaptive Immune Activation

Animal studies support associations between MNP exposure and mucosal barrier injury. In a mouse model, exposure to polyvinyl chloride (PVC) MNPs was associated with reduced intestinal mucus secretion (including reduced expression of mucus-related genes) and increased intestinal permeability compared with controls [32]. Another study reported that polystyrene MPs altered intestinal microstructure and were associated with decreased expression of tight junction-related genes alongside microbiome changes, suggesting a coupled dysbiosis–barrier phenotype [33]. Notably, environmentally aged MNP particles that have undergone UV degradation, oxidation, and surface fragmentation in real-world conditions exhibit altered surface chemistry and increased adsorption of toxic contaminants compared with the “pristine” particles used in most laboratory studies, potentially amplifying their pro-inflammatory and barrier-disrupting effects. These studies support mechanistic plausibility but specific variables in polymer type, particle morphology, dose metric, and exposure duration often differ substantially across experiments [34]. Additionally, “pristine” particles may not represent environmentally aged mixtures [34].

### 4.3. Translational Relevance to Chronic Inflammatory Disease

MNP exposure has also been linked to immune signaling changes in mice. In one study, mice fed varying levels of polyethylene microplastics displayed higher intestinal inflammation with increased pro-inflammatory signaling markers (including TLR4, AP-1, and IRF5), increased serum interleukin-1α, and alterations in CD4+ Th17 and Treg cell proportions [26]. Macrophage dysfunction is another proposed pathway; microplastics can induce oxidative stress, which may bias macrophage responses toward pro-inflammatory phenotypes and contribute to organ injury [35]. Immunoglobulin A (IgA), a key mucosal immune effector, has also been proposed as a potential biomarker of MNP-associated immune perturbation in some repeated-dose studies [36]. Collectively, these findings suggest that MNP exposure can engage multiple arms of the mucosal immune system, sustaining a pro-inflammatory state that may lower the threshold for chronic GI disease in susceptible individuals.

## 5. Microplastics, Dysbiosis, and Gastrointestinal Neoplasia

### 5.1. Mechanistic Links Between Microplastics and Carcinogenesis

GI neoplasia is increasingly included in MNP–GI discussions as carcinogenesis in the colon is strongly shaped by chronic inflammation, epithelial barrier integrity, microbial metabolites, and tumor immune regulation. These effects are plausible given the perturbation associated with MNP exposure [37]. A foundational cancer–microbiome hypothesis highlights multiple mechanistic routes (immune modulation, dysbiosis, genotoxicity, microbial metabolism) through which microbial ecosystems can promote or suppress tumor development, and MNPs are best conceptualized as potential upstream stressors that could influence those routes rather than as a single established carcinogen mechanism [37].

One key mechanism involves the induction of oxidative stress and inflammatory responses at the cellular and tissue levels. MNPs, and NPs in particular, can infiltrate cells and generate reactive oxygen species (ROS), which damage cellular components including DNA, proteins, and lipids. This oxidative damage can lead to DNA mutations and genomic instability, setting the stage for carcinogenesis [38]. Additionally, MNPs have been associated with chronic inflammation characterized by tissue injury, fibrosis, and altered immune responses. Chronic inflammation is a well-established driver of tumor initiation and progression by modifying the tumor microenvironment, promoting angiogenesis, and impairing immune surveillance [39].

MNPs also disrupt cellular signaling pathways and endocrine function. They act as endocrine disruptors by adsorbing and carrying toxic chemicals such as heavy metals, flame retardants, and plasticizers. These products can interfere with hormone receptors and signal transduction pathways that regulate cell proliferation and apoptosis. This hormonal dysregulation may facilitate uncontrolled cellular growth and tumorigenesis [37,40]. Moreover, MNP particles can modulate epigenetic mechanisms, altering gene expression profiles in ways that favor transformation and cancer development [41]. This area, however, requires more focused research.

In one of the few studies exploring the relationship between malignancy and NPs specifically, Kim et al. demonstrates that prolonged exposure of colorectal cancer cells (HCT116) to polystyrene NPs resulted in significant alterations in gene expression [42]. These effects included elevated expression of migration markers associated with cell motility, upregulation of epithelial–mesenchymal transition signaling, and cancer stem cell-associated gene signatures. These findings suggest specific consequences of NP exposure that may promote metastatic potential in colorectal cancer cells.

Furthermore, MNPs may alter microbiome and immune function. Their physicochemical properties can disrupt the host microbiota, which plays a crucial role in maintaining immune homeostasis and cancer prevention. Dysbiosis induced by MNPs can lead to immune modulation that favors tumor growth and metastasis [39]. The immune system’s reduced capacity to detect and eliminate emerging tumor cells in the presence of microplastic-induced chronic inflammation and oxidative stress creates a microenvironment conducive to tumorigenesis [39].

### 5.2. Emerging Human Evidence

Human data now extends beyond mere detection, but also towards disease association. In a large cohort study of IBD and controls, fecal microplastic concentrations were higher in IBD patients, with correlation to severity [25]. In 2025, a case–control study recruiting colorectal cancer (CRC) cases and controls from Quzhou People’s Hospital quantified fecal MNPs using laser infrared imaging and reported higher median fecal microplastic concentrations in CRC cases than controls, with a strong exposure–response in adjusted models [27]. These results are hypothesis-generating rather than causal, and careful confounding control is essential because diet and food packaging behaviors correlate with multiple CRC risk factors (diet quality, obesity, exposures) [27].

### 5.3. Preclinical and Mechanistic Evidence

Inflammation-associated CRC models provide mechanistic plausibility that MNPs can act as tumor promoters in an already inflamed colon. A mouse model of colitis-associated CRC reported greater tumor burden in mice receiving microplastics compared to controls [43]. A similar study focused on polystyrene NPs in mouse models reported increased colitis-associated CRC progression with evidence consistent with lipid metabolism disruption, oxidative stress/DNA damage, and PI3K/AKT/mTOR pathway activation [28]. Recent studies on microplastics isolated from tumor tissue and blood from patients with CRC proposed that tumor-infiltrating MPs can disrupt a JAK–STAT–microbiota axis, promoting immunotherapy resistance in CRC models, raising a clinically salient hypothesis linking environmental particulate exposure to treatment response heterogeneity [29].

Overall, MNPs may influence the risk and progression of CRC through a multitude of methods including oxidative stress and inflammation, intestinal barrier damage, immune dysregulation, endocrine disruption, and dysbiosis, as these mechanisms all contribute to a pro-carcinogenic state. Further research is critical to fully elucidate these pathways, establish exposure thresholds relevant to human health, and develop targeted interventions [38,39,40,41].

## 6. Vulnerable Populations and Disease Contexts

### 6.1. Early Life

Given that feeding practices and food-contact materials can generate high MNP particle loads, an emphasis on early life is paramount. Polypropylene infant feeding bottles can release microplastics at very high particle counts per liter during formula preparation, implying potentially high ingestion in infants compared with adults [7]. Pediatric stool studies provide further context: a pilot study of preschool children quantified stool microplastics and reported associations between MNP exposure and gut microbiota differences [23]. A larger multicenter study subsequently linked exposure to microbial profiles and antibiotic resistance pathways [44]. Emerging evidence suggests that MNP exposure may begin before birth. MNPs have been detected in placental tissue [45]. This raises concern that these particles, or their associated chemicals, may cross the maternal–fetal interface and influence fetal development, as well as immunologic and metabolic functions [46].

### 6.2. Inflammatory Bowel Disease

Barrier disruption, dysbiosis, and immune activation are central to IBD pathophysiology, making IBD a plausible susceptibility context for microplastic effects or retention. A large human population study reported higher fecal microplastic concentrations in IBD with correlation to severity, though causality could operate in either direction (exposure contributing to disease vs. disease increasing retention) [25]. Given that the intestinal barrier is a key regulator of inflammation and cancer risk, studies should specifically test whether barrier-impaired hosts exhibit increased MNP retention or greater inflammatory response at comparable exposures [31]. Additional emerging work reports that MNPs in fibrotic intestinal tissue and adjacent mesenteric adipose tissue in Crohn’s disease (CD) patients correlates to fibrosis severity, which could motivate studies of particle retention, translocation, and local immune–fibroblast interactions in IBD stricturing phenotypes [30].

### 6.3. Dietary Pattern Interactions

**Figure 3:**
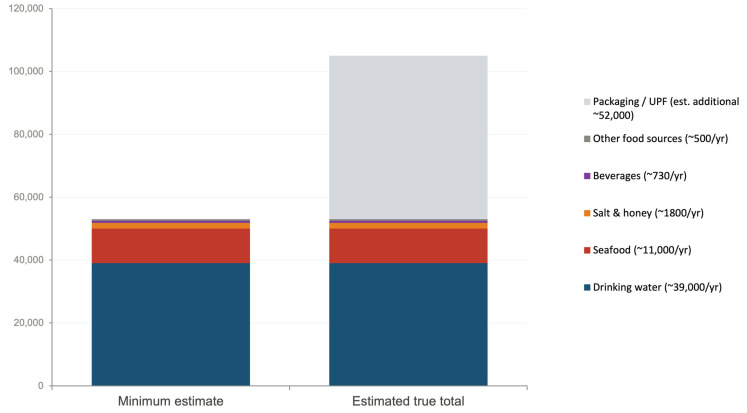
Estimated annual dietary MNP ingestion by source. Left bar: minimum estimate of approximately 52,000 particles per year based on [3] using reported consumption volumes and contamination levels across food categories. Right bar: estimated true total incorporating additional contributions from plastic packaging degradation and ultra-processed food sources, which remain incompletely characterized in the literature. Sources: Cox et al. [3]; Hayder et al. [47].

Diet is a primary determinant of MNP ingestion, and comparative analyses of contamination levels across food categories reveal a hierarchy of exposure risk that is directly relevant to IBD. Drinking water represents the largest contributor to annual MNP burden by volume, with reported contamination levels of approximately 10,000 particles per liter and estimated annual ingestion exceeding 39,000 particles based on typical daily consumption [47,48]. Seafood carries the highest per-gram burden, reaching up to approximately 15 particles per gram in shellfish due to ocean bioaccumulation, while salt and honey contribute approximately 400 and 600 particles per kilogram, respectively, reflecting concentration during processing. Beverages including beer and carbonated soft drinks carry approximately 100 particles per liter through packaging and processing contamination pathways. Processed and ultra-processed foods (UPFs) show the greatest variability, with multi-step plastic contact during industrial preparation generating unpredictable but often elevated contamination [30,46]. These figures contextualize the estimate that dietary MNP ingestion reaches at least 52,000 particles annually, with true totals likely substantially higher given incomplete characterization of UPF and packaging-derived sources (Figure 3) [3].

The foods carrying the highest MNP burdens are disproportionately consumed at elevated rates by patients with IBD. A recent prospective longitudinal study by Mayorga et al. of 198 adults (49 CD, 49 ulcerative colitis (UC), 100 healthy controls), incorporating shotgun metagenomics and causal mediation analyses, documented that both CD and UC patients reported significantly higher consumption of packaged fruit juices and soft drinks, and markedly lower intake of vegetables, fruits, and nuts compared with healthy controls—a dietary pattern corresponding closely to the higher-MNP-burden food categories [30,46,49]. The study further demonstrated that patients with IBD exhibited lower microbiome alpha diversity and higher dysbiosis scores relative to controls, with CD showing the most pronounced disruption. It also found that dietary quality indices correlated positively with alpha diversity and negatively with dysbiosis [47]. These observations are mechanistically consistent with the aforementioned established effects of MNP exposure on microbiome composition, suggesting that MNP co-exposure and poor dietary quality act through convergent mechanisms on the same microbial substrates rather than as independent risk factors.

Causal mediation analyses in the Mayorga cohort identified disease-specific microbiome pathways through which diet modulates GI inflammation. In CD, higher dietary quality and consumption of coffee and whole wheat bread were associated with lower Harvey–Bradshaw Index (HBI) scores through shifts in specific SCFA-producing taxa, including increased *Butyricimonas paravirosa*, *Odoribacter splanchnicus*, *Bacteroides thetaiotaomicron*, and *Lawsonibacter asaccharolyticus*, alongside reductions in *Bacteroides fragilis* and *Escherichia coli* [47]. The hexitol fermentation to acetate pathway accounted for approximately one-third of coffee’s total anti-inflammatory effect on HBI, while the proinflammatory effect of soft drink intake on HBI was mediated through a mixed-acid fermentation pathway accounting for over 80% of the observed effect—providing quantitative mechanistic support for the link between ultra-processed beverage consumption, microbiome disruption, and clinical disease activity [47]. In UC, Mediterranean-type diets, fruits, olive oil, and coffee reduced CRP and fecal calprotectin primarily through enhanced microbial richness and reduced dysbiosis rather than taxon-specific shifts, with the acetylene degradation pathway accounting for approximately 60% of the Mediterranean diet’s anti-inflammatory effect on CRP [47]. Given that MNP exposure consistently reduces alpha diversity and promotes dysbiosis in experimental models, and that these are precisely the microbial parameters mediating dietary protection in UC, it is mechanistically plausible that chronic MNP co-ingestion may attenuate the benefits of otherwise protective dietary patterns [16,18].

**Figure 4:**
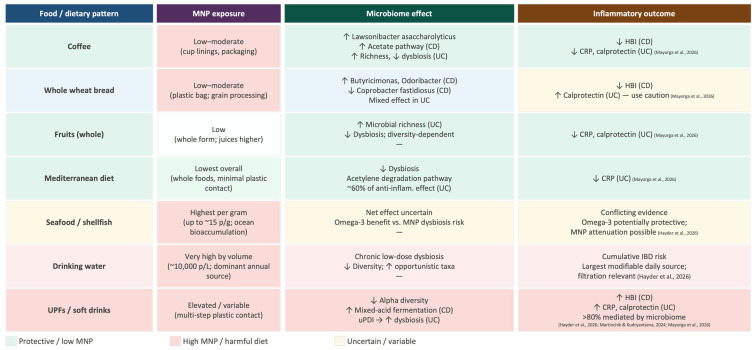
Food-specific MNP exposure burden, associated gut microbiome effects, and IBD inflammatory outcomes. Green shading indicates protective dietary patterns associated with low MNP burden; red/pink shading indicates harmful dietary patterns or high MNP exposure; yellow shading indicates uncertain or variable evidence. ↑ indicates increase or upregulation; ↓ indicates decrease or downregulation. Sources: Hayder et al. [47]; Martinchik & Kudryavtseva [48]; Mayorga et al. [49]; Cox et al. [3].

These findings collectively support a model in which diet shapes GI inflammation through two interacting pathways: the nutritional content of food, which determines microbiome community structure and SCFA-producing capacity; and the MNP burden of food delivery and preparation, which may degrade the same microbial substrates through which dietary benefit operates (Figure 4). Dietary patterns associated with the lowest MNP exposure—whole foods, minimal plastic-contact preparation, and Mediterranean-type diets—correspond closely to those associated with the greatest microbiome diversity and reduced inflammatory signaling in IBD. Prospective studies incorporating paired fecal MNP quantification alongside dietary quality indices, microbiome profiling, and inflammatory biomarkers are needed to formally test whether MNP burden modifies the strength of diet-mediated microbiome effects in CD and UC, and to establish whether exposure reduction constitutes a meaningful adjunct to dietary intervention in IBD management.

## 7. Methodological Challenges and Knowledge Gaps

Accurate quantification of MNPs in the human GI tract is a major methodological hurdle. Common analytical techniques, including Raman and µ-FTIR microspectroscopy, pyrolysis-GC/MS, and fluorescent dye imaging, each have limitations in detectable particle-size range, susceptibility to contamination, and polymer identification accuracy [50]. A further limitation across preclinical studies is the use of exposure doses and conditions that may not reflect realistic human dietary exposure; most animal and in vitro models employ acute, high-dose exposures with pristine particles, whereas chronic low-dose exposure to environmentally aged particle mixtures is the most clinically relevant scenario. Translating experimental findings to human risk estimates therefore requires caution, and studies using environmentally realistic concentrations and particle types are a priority [34]. For example, sample digestion and filtration steps can introduce or lose particles, and overlapping spectra can lead to polymer misclassification. Stool-based analyses provide a practical, noninvasive index of luminal exposure (e.g., multiple studies have measured MPs in human stool), and have been used to correlate fecal microplastic burden with IBD status [5]. However, by definition stool measurements do not capture particles retained in the mucosa or those that have translocated into tissues or blood. Tissue-based analyses (e.g., from biopsies or surgical specimens) and advanced imaging capable of detecting nanoscale plastics are technically demanding and are not yet standardized. A major barrier to progress is the lack of harmonized reference materials (standard particles with defined size distributions and polymer types) and unified reporting metrics across laboratories [5,51]. Without consensus standards and rigorous quality controls, results cannot be reliably compared across studies. Establishing agreed-upon particle standards, procedural blanks, and reporting guidelines will be essential for reproducibility, cross-study comparison, and the development of clinically meaningful exposure biomarkers [50].

The understanding of NPs and their physiologic impact as unique from MPs also deserves further attention. The conceptual framework of current studies often groups together MPs and NPs, which may result in collective interpretations of their effects. However, as discussed, MPs and NPs have distinct toxicokinetic properties resulting in differences in cellular uptake, systemic distribution, and physiologic clearance. The study of NPs in itself also poses specific challenges as they are substantially undercharacterized due to their small particle size, detection limits, and limitations of current technologies [52]. Further research specifically evaluating the differences between MPs and NPs is necessary to better understand their individual effects on gastrointestinal health.

## 8. Future Directions and Translational Implications

High-yield translational priorities include: (i) longitudinal IBD cohorts with paired fecal MNP quantification, calprotectin, and microbiome multi-omics; (ii) interventional exposure-reduction studies (e.g., reduced plastic heating/packaging) with stool and inflammatory markers; (iii) human-relevant models (exposed to environmentally realistic, real-world particles; and (iv) harmonized reporting to enable meta-analysis [53].

Finally, policy relevance should be framed carefully. Global plastic production and waste trends suggest ongoing exposure pressure, but clinical recommendations must remain cautious and evidence-aligned until dose–response relationships and intervention benefits are demonstrated [54].

## 9. Conclusions

The current evidence base supports biological plausibility that dietary MNP exposure can modulate GI health through microbiome disruption, barrier impairment, and inflammatory signaling, with early human biomonitoring and association studies offering hypothesis-generating signals for IBD and CRC. Mechanistic studies demonstrate that MNP exposure reduces microbial diversity, depletes SCFA-producing taxa, disrupts tight junction and mucus biology, and activates innate and adaptive immune pathways, with effects that vary by polymer type, particle size, aging state, and exposure dose. Human data now extend beyond detection to disease association, with higher fecal microplastic burdens reported in IBD patients correlated with disease severity, and elevated concentrations identified in CRC cases relative to controls. Preclinical models further support a tumor-promoting role in the context of established colonic inflammation.

Nevertheless, definitive human risk assessment and clinical guidance remain premature without standardized measurement, realistic exposure modeling, and stronger longitudinal or interventional human evidence. Prospective studies incorporating paired fecal MNP quantification, dietary quality indices, microbiome profiling, and inflammatory biomarkers are critical to clarifying causality and establishing whether dietary exposure reduction constitutes a meaningful adjunct to IBD and CRC prevention strategies.

## Figures and Tables

**Table 1:** Summary of key human, animal, and in vitro studies examining micro- and nanoplastic (MNP) exposure and gastrointestinal health outcomes. Studies are organized chronologically and include human biomonitoring, in vitro models, animal studies, and clinical association studies. ↑ indicates increase or upregulation; ↓ indicates decrease or downregulation. MNP = micro/nanoplastic; MP = microplastic; IBD = inflammatory bowel disease; CRC = colorectal cancer; AOM/DSS = azoxymethane/dextran sodium sulfate colitis-associated cancer model; CD = Crohn’s disease; UC = ulcerative colitis.

Author (Year)	Study Design	MNP Exposure	Key Findings	Limitations
Schwabl et al. [5]	Prospective case series	Human stool analysis	Detected up to 9 MP polymer types in human stool; first large human biomonitoring study; median 20 MP/10 g stool	No matched controls; no disease correlation
Lu et al. [16]	Animal model (mouse)	Polystyrene MPs, varying sizes	Gut microbiome dysbiosis with ↓ Firmicutes and ↓ α-Proteobacteria; reduced diversity by RNA sequencing of cecum	Animal model; pristine particles only
Nissen et al. [18]	In vitro colon model	PE and PS microplastics	↑ Enterobacteriaceae, Desulfovibrio, Clostridium; ↓ beneficial flora after single exposure	In vitro; single acute exposure
Yan et al. [25]	Human cross-sectional	Fecal MP quantification	↑ fecal MP burden in IBD vs. controls; burden correlated with disease severity	Cross-sectional; directionality unclear
Li et al. [26]	Animal model (mouse)	Polyethylene MPs	↑ intestinal inflammation; ↑ TLR4, AP-1, IRF5; ↑ serum IL-1α; altered CD4+ Th17/Treg proportions	Single polymer; high dose
Ibrahim et al. [14]	Human tissue analysis	Colectomy specimens	Microplastics detected and characterized in human colon tissue; supports tissue-level exposure	Small N; contamination control challenges
Xu et al. [27]	Case-control study	Laser IR imaging of fecal MPs	↑ median fecal MP in CRC cases vs. controls; dose-response in adjusted models	Single-center; confounding by diet difficult to exclude
Liu et al. [23]	Multicenter cross-sectional	Fecal MP; metagenome sequencing	MP exposure linked to altered microbiome composition and ↑ antibiotic resistance gene activity in preschool children	Cross-sectional; causal inference limited
Tian et al. [28]	Animal model (AOM/DSS)	Polystyrene nanoplastics	↑ colitis-associated cancer progression; lipid metabolism disruption; oxidative stress/DNA damage; PI3K/AKT/mTOR activation	Animal model; may not reflect human exposures
Jiang et al. [29]	Preclinical/translational	Tumor-infiltrating MPs from CRC patients	MPs disrupt JAK-STAT-microbiota axis; promote immunotherapy resistance in CRC models; clinically salient hypothesis	Mechanistic study; human causality unproven
Wen et al. [24]	Animal model (mouse)	Environmentally relevant MP concentrations	Cholestasis; impaired bile acid synthesis; altered bile acid efflux genes; abnormal bile acid concentrations in stool	Animal model; bile acid human relevance unclear
Wu et al. [30]	Human tissue (Crohn’s disease)	Surgical specimens + mesenteric fat	MPs detected in fibrotic intestinal tissue and adjacent mesenteric adipose; correlation with fibrosis severity	Small cohort; contamination control needed

## Data Availability

No new data were created or analyzed in this study. Data sharing is not applicable to this article.
